# Optimizing *In Situ* Vaccination During Radiotherapy

**DOI:** 10.3389/fonc.2021.711078

**Published:** 2021-10-26

**Authors:** Sayeda Yasmin-Karim, Jana Wood, Johanna Wirtz, Michele Moreau, Noella Bih, William Swanson, Ashley Muflam, Victoria Ainsworth, Bashkim Ziberi, Wilfred Ngwa

**Affiliations:** ^1^ Department of Radiation Oncology, Dana Farber Cancer Institute, Boston, MA, United States; ^2^ Department of Radiation Oncology, Brigham and Women’s Hospital, Boston, MA, United States; ^3^ Department of Radiation Oncology, Harvard Medical School, Boston, MA, United States; ^4^ Department of Immunology and Microbiology, University of Veternary Medicine and Pharmacy in Kosice, Kosice, Slovakia; ^5^ Medical Faculty, University of Ulm, Ulm, Germany; ^6^ Department of Physics and Applied Physics, University of Massachusetts Lowell, Lowell, MA, United States; ^7^ Department of Radiation Oncology and Molecular Radiation Sciences, Johns Hopkins University, Baltimore, MD, United States; ^8^ Department of Library and Information Science, Rutgers University New Brunswick, New Brunswick, NJ, United States; ^9^ Department of Physics, University of Tetova, Tetova, North Macedonia

**Keywords:** radiotherapy, immunogenic biomaterials, abscopal effect, immunotherapy, cancer vaccine, dose-painting, prostate cancer, pancreatic cancer

## Abstract

Effective *in situ* cancer vaccines require both a means of tumor cell death and a source of adjuvant to activate local dendritic cells. Studies have shown that the use of radiotherapy (RT) to induce tumor cell death and anti-CD40 to activate dendritic cells can result in *in situ* vaccination in animal models. Here, investigations are carried out on potential strategies to enhance such *in situ* vaccination. Strategies investigated include the use of smart immunogenic biomaterials (IBM) loaded with anti-CD40 in different tumor types including immunologically cold tumors like pancreatic and prostate tumors. The use of downstream checkpoint inhibitors to further boost such *in situ* vaccination is also examined. Results indicate that the use of IBM to deliver the anti-CD40 significantly enhances the effectiveness of *in situ* vaccination with anti-CD40 compared with direct injection in pancreatic and prostate cancers (*p* < 0.001 and *p* < 0.0001, respectively). This finding is consistent with significant increase in infiltration of antigen-presenting cells in the treated tumor, and significant increase in the infiltration of CD8^+^ cytotoxic T lymphocyte into distant untreated tumors. Moreover, *in situ* vaccination with IBM is consistently observed across different tumor types. Meanwhile, the addition of downstream immune checkpoint inhibitors further enhances overall survival when using the IBM approach. Overall, the findings highlight potential avenues for enhancing *in situ* vaccination when combining radiotherapy with anti-CD40.

## Introduction

The metastasis of cancer cells to distant organs is dominantly responsible for the primary cause of cancer morbidity and mortality (1). Approximately 60% of cancer patients receive radiation treatment (RT) as a part of the treatment process (2). Apart from the combination of traditional cytotoxic chemotherapy with radiotherapy, limited progress has been made in the synergies of radiotherapy and other targeted therapies to cure metastatic cancers (2). *In situ* vaccination approaches that combine RT with immunoadjuvants have major potential for curative treatment of patients with cancer metastasis (3–5). One promising approach involves using RT with the immunoadjuvant (IA) anti-CD40 (3, 6, 7). CD40 is a TNF family member expressed on antigen-presenting cells (APCs) like dendritic cells, macrophages, and B cells and some cancer cells. When engaged with CD40L or with an agonistic antibody (anti-CD40), CD40 signaling leads to upregulation of NF-κB and production of IL-12 and other related cytokines (6, 7). Hence, anti-CD40 monoclonal antibody can be employed to increase activation of APCs, which play an important role in generating an effective anticancer immune responses (3, 6). Recent animal studies have demonstrated promise when combining RT with locally delivered anti-CD40 even for immunologically cold tumors like pancreatic cancer (8, 9). The approach has potential to be effective across different tumor types, since the neoantigens generated *in vivo* by RT are tumor/patient specific, and the anti-CD40 can act on APCs present in the tumor microenvironment without the need for the tumor to express CD40 as with invasive prostate cancer (3, 10, 11).

*In situ* drug delivery is also particularly attractive as it bypasses intravenous drug delivery barriers limiting drug penetration into tumors while also significantly reducing systemic toxicities which are currently a critical barrier to use of anti-CD40 and other IA (12, 13). Contemporaneous developments in smart *in situ* drug delivery technology such as smart radiotherapy biomaterials (11) have highlighted the potential for smart delivery of anti-CD40 and other IA to boost cancer treatment outcomes (14). Such smart radiotherapy biomaterials include immunogenic biomaterials (IBM), which possess the ability to provide image guidance during image-guided radiotherapy (IGRT) while sustainably delivering IAs to the tumor microenvironment (15, 16). *In situ* delivery with IBM also bypasses untoward systemic toxicities and affords sustained release due to gradual degradation of the biomaterial, thereby increasing the bioavailability of the IA for activating local APCs (3, 14, 17). Also immunogenic biomaterials which degrade to release the IA may make the immunosuppressive tumor microenvironment more immunogenic and could further enhance *in situ* vaccination compared with direct intratumoral delivery of IA (16–18). The use of IBM may further offer a viable pathway to clinical translation as they could simply replace currently used inert RT biomaterials (e.g., fiducials, beacons) (3, 14, 15) at no additional inconvenience to many cancer patients. Here, the IBM approach is examined in comparison with direct *in situ* delivery. For additional perspective, the IBM approach is investigated for multiple immunosuppressive cancer models which include, pancreatic adenocarcinoma, castration-resistant prostate cancer, and cervical cancer. Furthermore, the approach with IBM is investigated in combination with downstream checkpoint inhibitors, anti-PD1 and anti-CTLA4 (19).

## Materials and Methods

### Cell Lines and Cell Culture Materials and Antibodies

C57/BL6 background murine pancreatic ductal adenocarcinoma (PDAC) origin cell line Panc-O2 was obtained from the National Cancer Institute. Murine transgenic C57/BL6 background prostate adenocarcinoma TRAMP-C1 cell line was purchased from ATCC. Both cell lines were cultured in Dulbecco’s modified Eagle’s medium (DMEM) (Gibco, Waltham, MA, USA) with 10% fetal bovine serum (FBS) (Sigma, ST. Louis, MO, USA) and 1% penicillin/streptomycin (Invitrogen, Waltham, MA, USA). To generate aggressive castration-resistant prostate tumors, TRAMP-C1 cells were grown in androgen-deprived medium (DMEM with 10 nM flutamide with 10% dextran-coated charcoal-treated FBS) for 2 weeks following a standard protocol (20). These androgen-deprived TRAMP-C1 (AD-TRAMP-C1) cells were used to generate castration-resistant prostate tumors for this study. Mouse cervical cancer cell line TC-1, expressing HPV16 E6/E7 with C57BL/6 background, was kindly provided by Dr. T.C. Wu (Johns Hopkins Medical Institutions, Baltimore, MD, USA). TC-1 cells were cultured in RPMI-1640 media supplemented with 10% FBS, 1% penicillin/streptomycin, 1% sodium pyruvate, 1% nonessential amino acids, and 10 mmol/L HEPES. All cells were grown in a humified incubator at 37°C under 5% CO_2_ atmosphere. All experiments were performed using cells with passage numbers less than 30, and all injected cells were tested to be mycobacterium free to avoid any potential adjuvant effect. Monoclonal agonistic anti-CD40 (clone FGK4.5/FGK45, BioXell, Lebanon, NH, USA) was used for either direct injection or in IBM production in this study. Mouse anti-CD11b (dendritic cells), anti-CD4 (helper T cell), and anti-CD8 (cytotoxic T cell) were purchased from Abcam.

### Computed Tomography Imaging and Image-Guided Radiotherapy

A small animal radiation research platform (SARRP, Xstrahl, Inc., Suwanee GA, USA) was used for computed tomography (CT) imaging and IGRT. Mice were anesthetized with isoflurane vapor. CT images were taken first at 65 kVP energies. The CT images were used for a single-fraction IGRT with field size to encompass one of the implanted SQ tumor (planning treatment volume (PTV)) using 220 kVp, 13 mA, and 0.15 mm copper (Cu) filter. A dose of 5 Gy with single fraction was given for prostate and pancreatic cancers and single fraction of 6 Gy was given for cervical cancer.

### Immunogenic Biomaterials

IBMs were developed with polymer components that have been shown to enhance immunogenicity (14, 15): including poly(lactic-co-glycolic) acid (PLGA) (MW: 50–50 kDa), sodium alginate (ALG) powder, and acetone from Sigma-Aldrich. Another IBM component is monoclonal antimouse CD40 antibody (BioXcell, Lebanon, NH, USA). The Harvard apparatus (Harvard Bioscience, Holliston, MA, USA) and silicone tubing (ID 1/32″, Saint-Gobain Performance Plastics Laboratory Division, Williamsburg, MI, USA) were used for shaping the PLGA IBM. PLGA IBM were fabricated by mixing PLGA and acetone (100 mg:2 ml, respectively) to get a homogenous mix. A Harvard apparatus (Harvard Bioscience) was used to infuse a prepared mixture at a constant flow rate into the silicon tubing (Saint-Gobain Performance Plastics Laboratory Division) with an internal diameter (ID 1/32″) similar to that of currently used fiducials. The loaded silicon tubing was dried at 50°C for 48 hours and then cut into lengths of 3 mm as needed. The monoclonal antibody payload (20 µg) was added to the core and both ends were sealed with additional gel. For the cervical cancer study, IBM with ALG gel was used. The ALG powder (Sigma-Aldrich) was dissolved in phosphate-buffered saline (PBS, Gibco) by rigorous vertexing at room temperature at a final a concentration of 5 mg/ml and then mouse monoclonal antibody to CD40 (20 µg) was added and mixed thoroughly. Prior to administration, the mixture was filtered through 0.22-µm syringe filter. IBM was administered intratumorally as performed clinically for fiducials using clinical brachytherapy 18-gauge needles (IZI Medical Products, Owings Mills, MD, USA) with one injection per tumor under isoflurane anesthesia.

### Mouse and Tumor Models

C57BL/6NTac background 8–12-week-old wild (W^+/+^) male and female mice were purchased from Taconic mice. The animals were contained in groups of five in standard cages with free access to food and water and a 12-h light/dark cycle. All mice were adjusted to the animal facility for at least 1 week before experimentation. All possible parameters that may cause social stress, like group size, among the experimental animals were carefully checked and evaded. Animals were observed three times a week after cell implantation for any physical abnormalities. For subcutaneous tumor models, Panc-02 (2 × 10^5^ cells/tumor) suspended in PBS were implanted to create syngeneic pancreatic subcutaneous (SQ) tumor both mouse model. Generated castration-resistant TRAMP-C1 cells (1 × 10^6^ cells/tumor) suspended 1:1 in PBS:HC (high concentrated Matrigel) were injected subcutaneously in both flanks of the same background male mice to generate prostate tumors. For cervical cancer, TC-1 cell line (1 × 10^5^ cells/tumor) was injected subcutaneously in both flanks of the same background female mice. In all cases, an insulin syringe with 22-gauge size needle was used for subcutaneous tumor cell injection. All mice were maintained following IACUC-approved protocol (protocol number 15-040). All animal experiments were conducted in compliance with the guidelines and regulations set by the Institutional Animal Care and Use Committee (IACUC).

### Research Design

In murine cancer models, two SQ tumors were implanted in two flanks of one mouse where only one was treated. Following the treatment parameters established in previous study (16), a low dose, 5 Gy, of IGRT and/or an IA were directly injected intratumorally (20 µg of dAntiCD40) to one of the two implanted tumors. The treated tumor was designated as primary tumor, and the untreated tumor was designated as secondary/abscopal/metastatic tumor. Growth of both tumors (treated and untreated) was analyzed. Furthermore, metastatic spreading of the implanted tumors was observed by analyzing *ex vivo* lung tissue macroscopically and microscopically 8 weeks postimplant. Next, IA IBM was used intratumorally for sustained release of anti-CD40 as well as to increase the immunogenicity of the tumor tissue. Multiple cancer models were tested for IBM+RT. To further observe the immune response, check point inhibitors were added to the IBM+RT treatment regimen. In all cases, treatment outcome was analyzed by observing tumor growth and survival rate.

Tumor growth was supervised regularly per approved animal protocol. A digital Vernier caliper was used to measure the tumor length and width to calculate the volume. The length was measured along the imaginary longitude of the leg; the width was measured in the direction of the latitude. The tumor volume was calculated using the formula: Tumor volume = [1/2 * *L* * (*W* ^2^)] where *L* and *W* is the length and width of the tumor, respectively. When tumor volume reached to approximately 25–35 mm^3^, mice were then randomized into different cohorts for treatment. All treatments were given directly to one tumor either by direct intratumoral injection or by intratumoral implantation of IBM with or without RT. Intratumoral IBM was administered in one IBM/tumor using clinical brachytherapy needles as described above. A SARRP was used for IGRT. Tumor volumes were calculated for both tumors, on the day of treatment (day 0) and 2 times/week posttreatment by measuring the length and width of the tumor. A survival study was also performed. Mice were euthanized when either tumor exceeded 20 mm in diameter collectively and/or when any of the tumors became ulcerated or ruptured. A control cohort was created with no treatment (PBS only) and another cohort was created to administered IBM loaded with same amount of PBS only. The tumor volume was plotted against time. Animal study was performed following IACUC-approved protocol, which was predetermined based on published evidence justifying such a study design.

### Hematoxylin and Eosin Staining

Lung tissue in applicable cases were extracted and fixed in 10% formalin for 24 h. Paraffin-embedded tissues were sliced into 4-µm-thick sections with a microtome, air-dried, fixed with acetone, and stained accordingly following standard protocol. To observe lung metastasis, sections were stained with hematoxylin and eosin (H&E), and whole slide scanning (×40) was performed on an EasyScan infinity (Motic, Wetzlar, Germany). High-magnification images were collected using Case Viewer software.

### Immunofluorescence Assay

Sections stained with immunofluorescence were incubated overnight in a cold room with the antimouse primary antibody (as required), rabbit anti-CD11b (1:500, Abcam), rabbit anti-CD4/80 (1:500, Abcam), anti-CD4 (1:500, Abcam), or rabbit anti-CD8 (1:200, Cell Signaling Technology, Danvers, MA, USA). The sections were subsequently immunohistochemically stained with specific fluorophore-conjugated goat anti-rabbit (F-2765, 1:500, Invitrogen). Fluorophore FITC (488 nm excitation wavelength), CY3 (555 nm excitation wavelength), or CY5 (670 nm excitation wavelength) antibody. Sections were then incubated with DAPI, and the slides were scanned (×40) using a Panoramic Midi Scanner (3DHISTECH, Budapest, Hungary). High-magnification images were obtained using Case Viewer software, and intensity of fluorescence was measured using Fiji/ImageJ software following standard protocol. Corrected total fluorescence (CTF) was analyzed following the standard formula [CTF = Integrated density − (Area of selected cell × Mean fluorescence of background readings)], and average intensity was calculated.

### Statistical Analysis

Survival data were plotted, and statistical analyses were performed using GraphPad prism v7.0. The Kaplan-Meier statistics (Madsen 1986, Statistical Concepts, Prentice Hall, Englewood Cliffs, NJ, USA) was utilized. A log-rank test was employed to determine the *p*-value for the Kaplan-Meier curves. Further analysis of the mice survival was performed in RStudio (RStudio, Inc., Boston, MA, USA) using a competing-risk regression. For the hazard ratios, survival differences among the treatment groups were adjusted to the initial tumor volumes in a Cox regression model. The *p*-values for the hazard ratios were corrected using the Bonferroni method. Statistical analyses for tumor volume were achieved using two-way ANOVA: two factors with replication tool and standard Student’s two-tailed *t*-test. *p*-values <0.05 were considered significant. ^*^ *p* < 0.05, ^**^ *p* < 0.01, ^***^ *p* < 0.001, and ^****^ *p* < 0.0001 were considered statistically significant.

## Results

**Figure 1:**
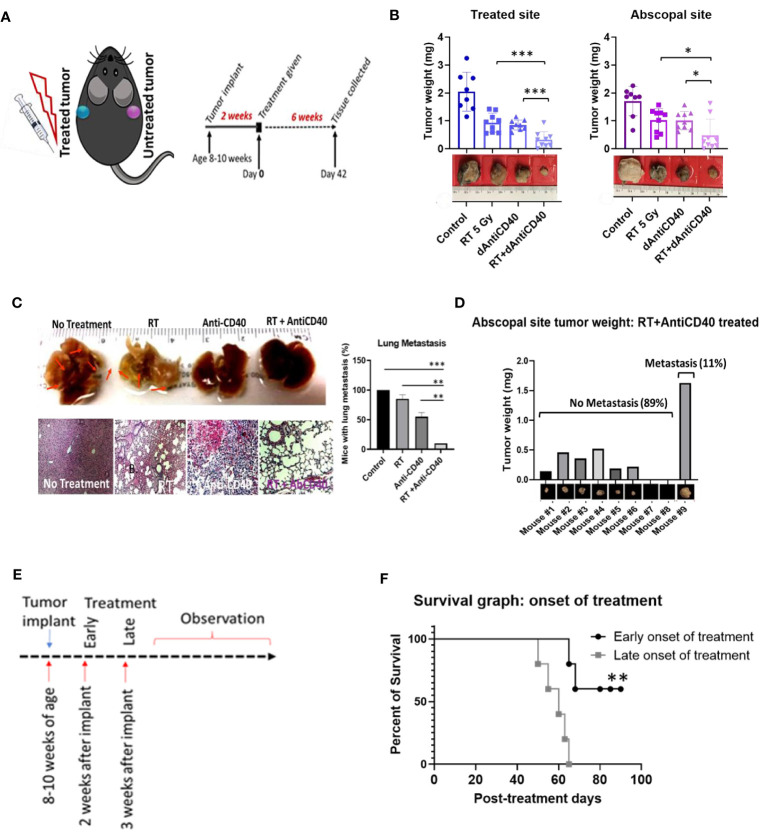
Anti-CD40+RT generates effective *in situ* vaccination (abscopal effect) and can prevent distant lung metastasis. **(A)** Cartoon showing mouse treatment model with two pancreatic tumors in two flanks where only one tumor was treated and experimental schedule of the study observed for 6 weeks posttreatment of 5 Gy of IGRT or dAntiCD40 (20 µg), or the combination (RT+dAntiCD40), along with a control. **(B)** Showing *ex vivo* tumor weight of treated and untreated secondary/abscopal tumors, 6 weeks posttreatment. Inset, pictures of the representative tumors. Here, the developed palpable sized one of the subcutaneous tumors was given 5 Gy of IGRT (*n* = 8) or dAntiCD40 (*n* = 8), or RT+dAntiCD40 (*n* = 9). Antimouse CD40 antibody was injected intratumorally with of 20 µg in PBS for a single time. The control group (*n* = 8) was injected with same volume of PBS. **(C)**. Macroscopic view (top) and hematoxylin and eosin-stained microscopic/histological view (bottom) of *ex vivo* lungs of the same group of mice, which were harvested 8 weeks postimplant (6 weeks posttreatment) and fixed in 10% formalin. Twenty-four-hour formalin-treated tumors were imaged for macroscopic pictures. For histological analysis, samples were imbedded in paraffin for tissue processing and generated 0.5-mm-thick slides. Scale bar represents 100 µm. A bar graph showing number of mice in each treatment group with microscopic lung metastases 8 weeks after tumor implantation. **(D)** Bar graph showing tumor weight of the abscopal response (untreated) tumors of the combination treated (RT+dAntiCD40) group corelating with presence of lung metastasis shown in histological analysis. **(E)** study design for the treatment timing showing early (2 weeks post implant) and late (3 weeks post implant) treatment onset with the combination treatment group (RT+dAntiCD40). **(F)**. Kaplan Meier survival graph for early and late treatment onset of combination treatment with RT+dAntiCD40 in pancreatic adenocarcinoma mouse model (*n* = 5). ^*^ *p* < 0.05, ^**^ *p* < 0.01, and ^***^ *p* < 0.001. Error bars are SD. ^***^ *p* < 0.001.

**Figure 1A** illustrates the treatment design with C57BL/6 syngeneic immune-competent mouse model. Two tumors of pancreatic adenocarcinoma (Panc-02 cell line) were implanted contralaterally in two flanks, as done in a previous study (16), where only one tumor was selected for the treatment as the primary tumor, and the distant untreated tumor was selected as a secondary metastasis for monitoring *in situ* vaccination. In the first study, the primary tumors were treated with one fraction of 5 Gy of IGRT (*n* = 8) or dAntiCD40 (20 µg) (*n* = 8), or the combination (RT+dAntiCD40) (*n* = 9). A cohort of mice with no treatment was selected as the control (*n* = 8). CT image-guided radiation treatment was provided to a single tumor using the SARRP for 5 Gy of RT dose. All tumors were resected 6 weeks posttreatment (8 weeks postimplant) to measure the weight. *Ex vivo* weight of the combination-treated tumors showed the highest reduction for both treated (primary) (*p* < 0.001) and the untreated secondary (metastatic) tumors (*p* < 0.05) compared with single treatments (**Figure 1B**), confirming the *in situ* vaccination.

Furthermore, this combination treatment curbed the formation of distant metastasis in the lungs. In analysis of the *ex vivo* lungs, 8 weeks after implantation of the tumors, no macroscopic was observed in the combination-treated mice. Additional histopathological analysis of the *ex vivo* lung tissue demonstrated absence of metastatic progression in 89% (*p* < 0.001) of cases compared with 100% of the control mice which developed metastasis. This compares with about 75% (*p* < 0.01) for RT only group and 50% (*p* < 0.01) for anti-CD40-only treatment groups, which developed lung metastasis (**Figure 1C**). Additional analysis shows that 89% of the mice which did not develop lung metastasis in the combination treatment group correspond with mice that showed robust *in situ* vaccination. Meanwhile, the other 11% which did not respond well developed lung metastasis (**Figure 1D**). In additional studies, Kaplan-Meier survival assay revealed that late onset of treatment is not as effective as early onset of the treatment (**Figures 1E, F** **)**, as would be expected. When the same combination treatment was given to a group in later stage, survival duration was significantly decreased compared with early onset of treatment (*p* < 0.001). This justifies investigations into further approaches to enhance *in situ* vaccination when using a single fraction of low dose RT with anti-CD40.

**Figure 2:**
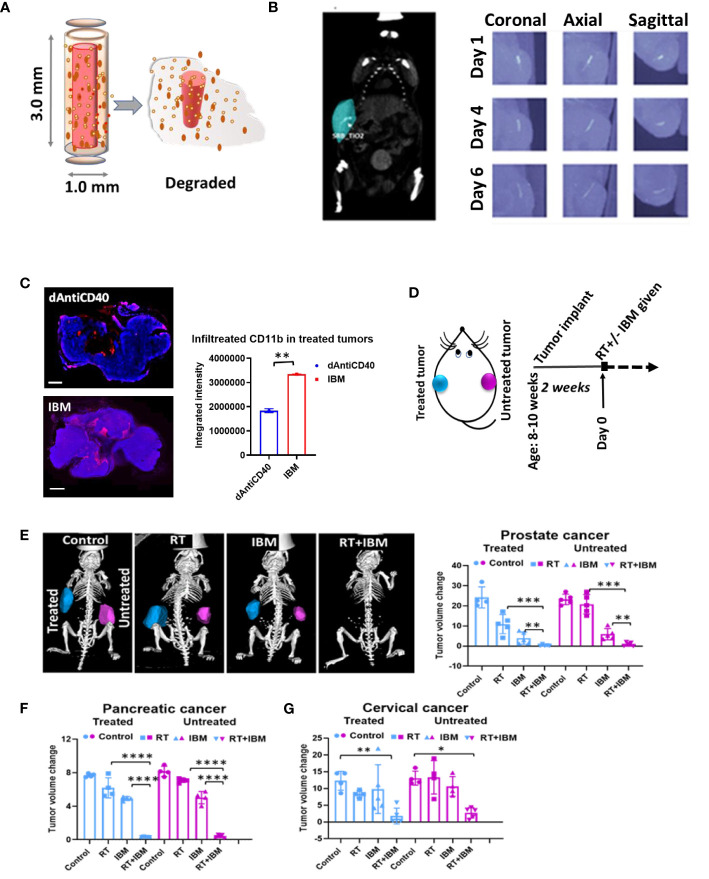
Anti-CD40 agonist-loaded immune-biomaterial (IBM) may provide imaging contrast and induces higher immunogenicity in treated tumor microenvironment. **(A)** Schematic diagram of IBM made of immunogenic polymer with antibody core (red), which releases as it biodegrades. **(B)** CT image showing IBM in tumor (blue color) highlighted using Imalytics software (left) and gradually fading CT images of IBM from days 1 to 6, as polymer biodegrades (right). **(C)** Fluorescent images and representative bar graph of the average fluorescent intensity of immunofluorescence-stained prostate cancer tissue treated with mouse CD11b^+^ antibody at posttreatment day 7. Corresponding immunofluorescence (merged) pictures showing infiltration of CD11^+^ dendritic cells (red) in tumor tissue. Cancer cell nucleus (blue, DAPI) when IBM or dAntiCD40 was given intratumorally (*n* = 3). Scale bar is 2,000 µm. **(D)** Treatment design and **(E)** prostate tumors generated from TRAMP-C1-derived castration-resistant prostate cancer cells treated with IGRT (5 Gy) and/or IBM, representing CT images (Imalytics software analyzed) of animals from different treatment cohorts with treated tumors (blue) and untreated tumors (pink). Bar graph represents treated and untreated tumor volumes on day 10 posttreatment (*n* = 5). **(F, G)** The tumor volumes of pancreatic (*n* = 5, IGRT 5 Gy^16^) and cervical cancer (*n* = 5, IGRT 6 Gy^29^), respectively 10 days after the same treatment regimen. Data represent the mean ± SD. *p < 0.5, ^**^ *p* < 0.01, ^***^ *p* < 0.001, ^****^ *p* < 0.0001.

In a further study, the potential of using IBM to sustainably deliver anti-CD40 in priming *in situ* vaccination was investigated as a possibility to enhance the effect of *in situ* vaccination. IBM are designed similar to fiducial markers (14, 16, 21), which are currently used during image-guided radiotherapy to ensure precision targeting of RT (**Figure 2A**). This can be directly administered into the tumor by 18-gauge brachytherapy needle (**Figure 2B**) for slow and sustained release of anti-CD40 (15, 16). In further investigation for tumor microenvironment, immunofluorescence staining for dendritic cell marker (CD11b) was performed on 7 days posttreated tumor tissue. A significant increase of CD11b^+^ dendritic cell (APC) infiltration was observed in the tumors treated with IBM compared with direct intratumor injection (dAntiCD40) (**Figure 2C**). This highlights the potential of IBM to make the tumor more immunogenic. Such infiltration and presence of APCs to pick up exposed antigens initiated by RT is critical for effective *in situ* vaccination.

Adding a single fraction of RT with IBM (**Figure 2D**), *in situ* vaccination was also observed for different tumor types including prostate (**Figure 2E**), pancreatic (**Figure 2F**), and cervical cancers (**Figure 2G**). Here, a single treatment with RT or IBM was compared with the combination treatment of RT+IBM where a low dose of IGRT was given in following doses: prostate (5 Gy), pancreatic (5 Gy), and cervical cancer (6 Gy). Twenty micrograms of anti-CD40 agonist was used in IBM. The control group received no treatment. The treatments were administered to only one of the bilaterally implanted flank tumors. All treatments were given to the tumors after reaching a volume of around 25–35 mm^3^, about 2 weeks after implantation. Remarkably, in prostate cancer, where tumor cells do not express CD40 (22) and capable of escaping the immune system by downregulating the class I APCs, like dendritic cell and macrophages (23), a combination treatment of 5 Gy of RT+IBM demonstrates major reduction of tumor volumes in androgen-deprived prostate cancers created by AD-TRAMP-C1 cells. Similar outcomes were also observed in pancreatic adenocarcinoma, and cervical cancer models confirming the combination treatment with RT+IBM may be a viable therapeutic strategy, across different cancer types, causing regression of the treated as well as the untreated distant metastatic tumors. In all cases, a single fraction of low-dose radiation was enough to generate the vaccine effect.

**Figure 3:**
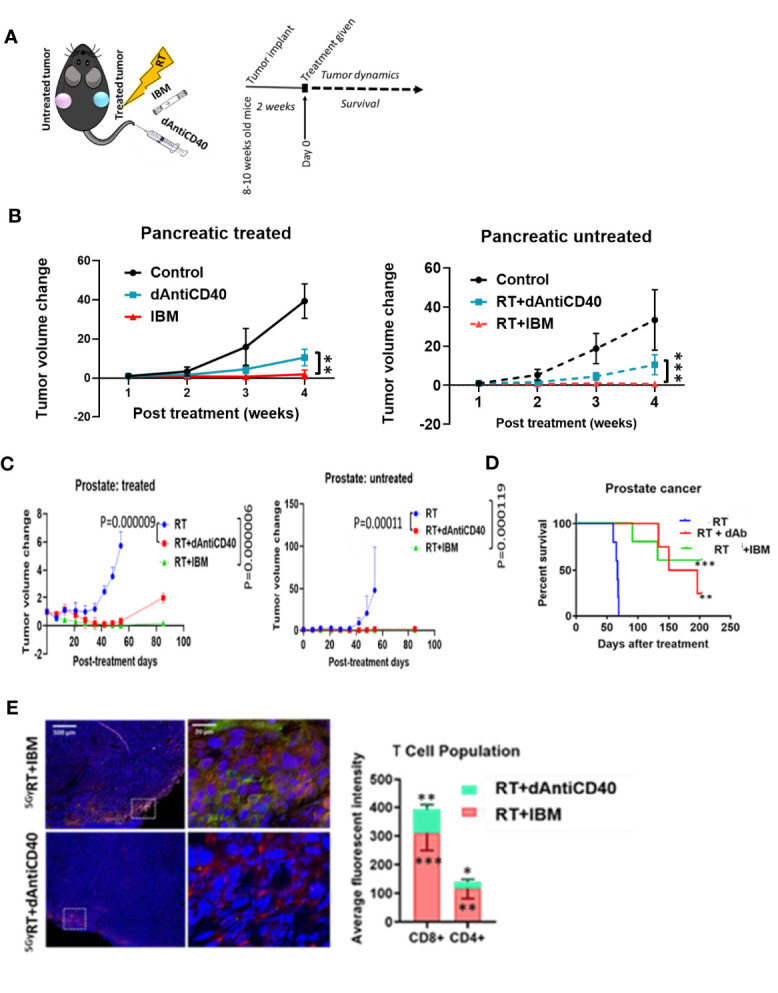
IBM enhances higher abscopal effect when compared with direct intratumor injection of anti-CD40 agonist (dAntiCD40). **(A)** Study design. **(B)** Line graphs showing dynamics of tumor volume change of pancreatic cancer for both treated (solid line) and the other untreated cancer (dashed line, *in situ* vaccination effect) where IGRT of 5 Gy was given either as direct intratumoral injection of anti-CD40 (dAntiCD40) or IBM along with 5 Gy of radiation. **(C)** In prostate cancer model, line graphs showing dynamics of tumor volume change in prostate cancers of both treated (left) and the other untreated/metastatic cancer (right *in situ* vaccination effect) where image-guided radiotherapy (IGRT) at 5 Gy was administered either in combination with direct intratumoral injection of anti-CD40 (dAntiCD40) or IBM. **(D)** Corresponding Kaplan-Meier survival graph. **(E)** Representative images and bar graph showing IF staining of untreated pancreatic cancer tissue stained with CD4^+^ and CD8^+^ antimouse antibody, showing intratumoral infiltration of CD4^+^ (green) and CD8^+^ (red) T lymphocytes, comparing the abscopal effect of tumors treated with RT+dAntiCD40 (*n* = 3) and RT+IBM (*n* = 3). In all cases, nucleus is stained with DAPI (blue). Scale bar represents 200 µm. The corresponding bar graph showing average fluorescence intensity of infiltrating helper CD4^+^ and cytotoxic CD8^+^ T lymphocytes in untreated tumor tissue on the opposite flank (abscopal tumors) 14 days following the treatment. Data represent the mean ± SD. ^*^ *p* < 0.5, ^**^ *p* < 0.01, ^***^ *p* < 0.001.

Additional analysis of study data focused on enhancing the effectiveness of *in situ* vaccination with anti-CD40 compared with results when anti-CD40 is delivered *via* IBM. Results focused on this are highlighted in **Figure 3**. The data show the use of IBM to deliver the anti-CD40 significantly enhances the effectiveness of *in situ* vaccination with anti-CD40 compared with direct injection (**Figure 3A**) in pancreatic (**Figures 3A, B**, *p* < 0.001) and prostate cancers (**Figure 3C**, *p* < 0.0001) with increase survival with *p* < 0.01 and *p* < 0.001, respectively (**Figure 3D**). The presence of T cells in RT+IBM-untreated group was also significantly higher compared with RT+dAntiCD40-untreated group (**Figure 3E**).

**Figure 4:**
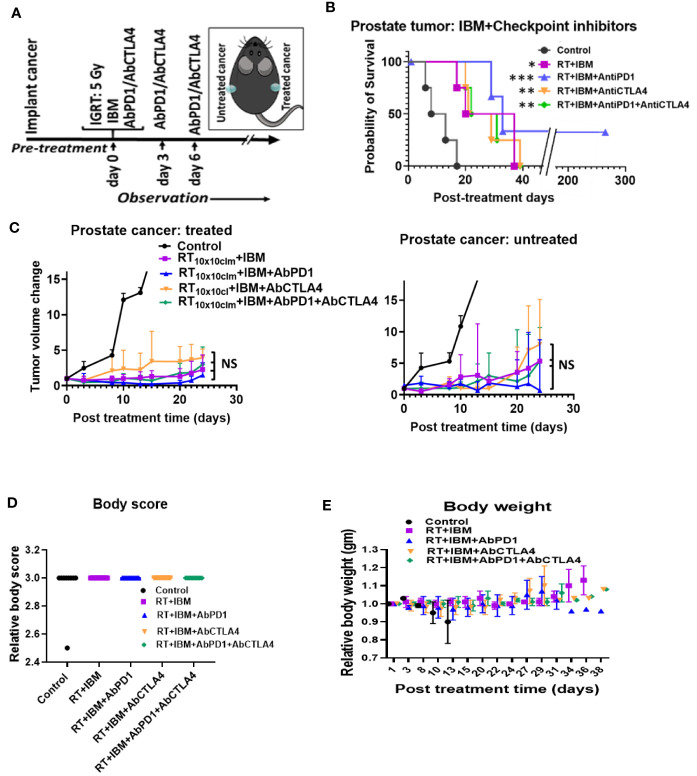
IBM further enhance the survival duration in combination with anti-PD1 and anti-CTLA4. **(A)** Research design to treat prostate cancers adding anti-PD1 and anti-CTLA4 antibody (IP) with the RT+IBM treatment. One treatment of IBM was given intratumorally (IT) in one out of two implanted tumors following 5 Gy IGRT in the same cancer on day 0. Anti-PD1and/or anti-CTLA4 was given intraperitoneally (IP) on days 0, 3, and 6. **(B)** Kaplan-Meier survival curve and **(C)** line graphs of dynamics of tumor volume change of both treated (left) and the other untreated/metastatic cancers (right) of this study with 5 Gy of IGRT+IT IBM followed by IP injection of anti-PDL-1 and/or CTLA4. **(D, E)** representing graph showing body score and body weight of the same cohort groups. All statistical significance was compared with controls. ^*^ *p* < 0.05, ^**^ *p* < 0.01, ^***^ *p* < 0.001; NS, not significant.

Despite becoming a standard of care for treatment of several cancers like melanoma, single-agent or dual checkpoint inhibitor therapy is not effective in cold cancers like prostate tumor (8, 9, 19). Such checkpoint inhibitors act downstream in the cancer immunity cycle to prevent tumor cells from blocking T-cell action following upstream priming action by APCs. There is strong rationale for combining the IBM approach with such immunotherapies to enhance *in situ* vaccination (19). Therefore, the potential for enhancing the IBM treatment approach with downstream checkpoint inhibitors, anti-PD1 and anti-CTLA4 was investigated (**Figure 4A**). Results from randomly treated tumors showed that for animals with high tumor burden, adding systemic (intraperitoneally (IP)) doses of anti-PD1, anti-CTLA4, and both together, respectively, improve the overall survival (**Figure 4B**) with no significant reduction of tumor volumes (**Figure 4C**), including body weight (**Figure 4D**) and body score (**Figure 4E**). For a more precise comparison, survival rates were adjusted to the initial pretreatment tumor size. The adjusted hazard ratios (HR) for each treatment group compared with control can be found in **Table 1**. For all tested treatment regimens, the chance to reach one of the previously stated endpoints was significantly lower than for the control group (HR <1, Bonferroni adjusted *p*-value <0.05), hence again all tested treatments were shown to prolong the survival period.

**Table 1:** Hazard ratios for each treatment using RT+IBM and additional checkpoint inhibitors compared with control adjusted to the initial pretreatment tumor size.

Treatment given	Hazard ratio	95% Confidence Interval	*p*-value adjusted
RT SGy+IT IBM	0.0096	[0.0006; 0.1641]	0.0094
RT SGy+IT IBM+IP anti-PD-1	0.0030	[0.0001; 0.0667]	0.0017
RT SGy+IT IBM+IP anti-CTLA-4	0.0061	[0.0003; 0.1102]	0.0038
RT SGy+IT IBM+IP anti-PD-1+IP anti-CTLA-4	0.0027	[0.0001;.0583]	0.0012

The lowest HR and therefore the greatest survival prolongation was achieved by adding both anti-PD1 and anti-CTLA4 to the IBM approach (HR: 0.0027, Bonferroni adjusted *p*-value <0.05). The next lowest HR was attained by adding anti-PD1 (HR: 0.0030, Bonferroni adjusted *p*-value <0.05). Adding anti-CTLA4 lowered the HR to 0.0061 compared with an HR of 0.0096 for the RT+IBM only group, which, nevertheless, still poses a significant prolongation of the survival (all Bonferroni adjusted *p*-values <0.05). These results show that adding systemic checkpoint inhibitors, like anti-PD1 and anti-CTLA4, along with IBM may facilitate the IBM-mediated enhancement of abscopal effects to further prolong the survival duration.

## Discussion

The use of smart radiotherapy biomaterials (14–17) was recently proposed as a novel approach to overcome immunosuppresion and toxicity limitations of the abscopal effect (3, 9). Here, we used IBM as an immunoadjuvant to enhance the abscopal effect in different cancer models where a low dose of IGRT was used for the radiation therapy. The results in this study provide experimental evidence corroborating the theraputic benefit of this approach across different tumor types including immunologically cold tumors (8) especialy in pancreatic and prostate cancers. Even though differences between the therapeutic effects existed among different cancer models and between primary and abscopal tumors, the results show that this treatment approach consistently boosts abscopal response rates across different tumor types.

The results in this study provide experimental evidence corroborating the theraputic benefit of this approach across different tumor types including the immunologically cold tumors of pancreatic and prostate cancers. Prostate cancer is one of the most diagnosed malignancies among men worldwide and remains the second leading cause of cancer-related death in the USA (24). For patients with localized prostate cancer, treatment options include surgery or radiotherapy, with concomitant or subsequent use of androgen deprivation therapy (ADT). Generally, PSA level should be <0.5 ng ml^−1^ after radiotherapy and <0.2 ng ml^−1^ after a radical prostatectomy (24), and occurrence of two consecutive PSA level elevations is often considered biochemical recurrence or progression. Biochemical recurrence develops in about 10% of low-risk and up to 60% of high-risk prostate cancer patients after external beam radiotherapy and in 20%–30% of patients after radical prostatectomy, despite use of ADT (24–26). For patients who present with advanced (metastatic) prostate cancer, ADT is the mainstay treatment (26). Despite initial responses, almost all patients progress to androgen-resistant metastatic disease, like metastatic castration-resistant prostate cancer (MCRPC), which is the main cause of death (26). Recently, there are increasing calls for innovation (26) in developing new therapy options or strategies for patients with MCRPC, whose treatment options are limited and prognosis poor. During external beam prostate radiotherapy, inert radiotherapy biomaterials: fiducial markers or beacons are administered to provide image guidance during treatment. The results in this study motivate further clinical translation studies where such inert biomaterials can simply be replaced with IBM. In this study, we also demonstrate how adding checkpoint inhibitors to the IBM approach once again may improve the IBM-mediated enhancement of abscopal effects and survival durations. The treatment with RT and the local administration of anti-CD40 *via* IBM with or without additional checkpoint inhibitors has great potential for the therapy of patients with MCRPC.

Meanwhile, for pancreatic cancer, with a dismal 5-year survival rate of 8% (27, 28), the potential of leveraging *in situ* vaccination to treat metastasis is crucial, given that most pancreatic cancer patients are diagnosed already with metastatic disease, with limited treatment options. The results using RT+IBM justify further studies here. An advantage of the IBM here is the ability to use small amounts of immunoadjuvant payload which provides a minimal toxicity advantage, which have hampered immunotherapy efforts for pancreatic cancer (14).

Currently, inert biomaterials (fiducials/spacers) are routinely implanted in the clinic to ensure spatial accuracy during radiotherapy and reduce the margins for the planning target volume (21). The use of IBM in place of fiducial markers as proposed with the IBM approach provides a viable pathway to clinical translation at no additional inconvenience to cancer patients. Besides this, the use of IBM for sustained *in situ* delivery of payloads (29) is a relatively more convenient way to deliver the immunoadjuvants for patients compared with repeated injections even if done intratumorally. The potential of using fewer or even single fractions of RT would also be a major convenience for cancer patients who often need to come in many times for treatment. Such hypofractionation would help reduce healthcare costs, increase access to treatment, and reduce disparities in the USA and around the world, as highlighted in our recent work (30). The IBM employed in this study also have the potential for CT imaging contrast (16, 17). Inclusion of high-Z nanoparticles can enhance image contrast and provide both CT and MRI imaging (15, 17). Despite recent data indicating that prostate cancer incidence is declining, the overall prostate cancer-related mortality continues to rise among African-American men (30). African-American men are more often diagnosed with metastatic prostate cancer compared with any other racial/ethnic groups (30, 31). Also, cervical cancer is a dominant cause of cancer-related death in low- and middle-income countries (29). Such reality provides impetus for further studies on approaches such as the RT+IBM approach.

CD40 expression is found in the tumor microenvironment, in APCs including dendritic cells, B cells, macrophages, and monocytes and in some solid tumor cells including lung, breast, kidney, and bladder (9, 10, 32, 33). Some immunologically cold tumors like prostate and pancreatic cancers show very low expression of CD40 in their microenvironment (10) and foreseeably, very rarely respond to the checkpoint inhibitors (9, 10, 34, 35). A key goal of immunomodulatory approaches for cold tumors is to establish inflammation in the tumor microenvironment (9). Ionizing radiation can have important immune-modulatory effects at the right dose and schedule. Radiotherapy can induce DNA damage response and immunogenic cell death, stimulating an antitumor inflammatory microenvironment to generate *in situ* vaccination (9, 10). Ionizing radiation can also be synergistic with immunotherapy. For example, radiotherapy increases the local secretion of interferons like IFN-γ, resulting in the upregulation of MHC-I in APCs, rendering them more susceptible to T-cell attack (5, 35). Fortifying the tumor microenvironment with CD40-ligand/anti-CD40 agonist may further enhance the antitumorigenic effect of checkpoint inhibitors. This phenomenon was observed in this study by further increasing the survival duration of the combination-treated mice with added anti-PD1 or with both anti-PD1 and anti-CTLA4. Moreover, it is always possible that the anti-CD40 ends up in other locations, and further pharmacokinetics and pharmacodynamics studies are needed to clearly established this. An extensive investigation in the molecular level may reveal details about the underlying mechanism of this treatment regimen for future clinical trials.

Overall, in this study, we demonstrate that the use of IBM with IA-like anti-CD40 can significantly enhance *in situ* vaccination during radiotherapy across different cancer models. The findings also highlight potential for enhancing such *in situ* vaccination with downstream checkpoint inhibitors. The results provide impetus for further studies towards clinical translation of this approach. This treatment approach with use of single or few radiotherapy fractions could lead to significant reduction in treatment time and costs, which also can have impact in reducing global health disparities (36).

## Data Availability Statement

The raw data supporting the conclusions of this article will be made available by the authors, without undue reservation.

## Ethics Statement

The animal study was reviewed and approved by the Dana-Farber Institutional Animal Care and Use Committee (IACUC), Dana Farber Cancer Institute, Boston, MA, USA.

## Author Contributions

SY-K designed the work, acquired and analyzed data, and wrote the manuscript; JaW, JoW, and WS, acquired and analyzed the data. MM, NB, AM, VA, and BZ analyzed the data. All authors revised the manuscript. WN contributed to the concept of the work, designed the work reviewed, and revised the manuscript. All authors contributed to the article and approved the submitted version.

## Funding

This research was supported by a National Institutes of Health (NIH) grant titled, “Combining radiotherapy and immunotherapy using next-generation radiotherapy biomaterials” under Award number 1 R21CA205094-01A1 and R01CA239042. The content is solely the responsibility of the authors and does not necessarily represent the official views of the NIH.

## Conflict of Interest

The authors declare that the research was conducted in the absence of any commercial or financial relationships that could be construed as a potential conflict of interest.

## Publisher’s Note

All claims expressed in this article are solely those of the authors and do not necessarily represent those of their affiliated organizations, or those of the publisher, the editors and the reviewers. Any product that may be evaluated in this article, or claim that may be made by its manufacturer, is not guaranteed or endorsed by the publisher.

## References

[1] Seyfried TN Huysentruyt LC. On the Origin of Cancer Metastasis. Crit Rev Oncog (2013) 18(1-2):43–73. doi: 10.1615/critrevoncog.v18.i1-2.40 23237552 PMC3597235

[2] Sharma RA Plummer R Stock JK Greenhalgh TA Ataman O Kelly S. Clinical Development of New Drug-Radiotherapy Combinations. Nat Rev Clin Oncol (2016) 13(10):627–42. doi: 10.1038/nrclinonc.2016.79 27245279

[3] Ngwa W Irabor OC Schoenfeld JD Hesser J Demaria S Formenti SC. Using Immunotherapy to Boost the Abscopal Effect. Nat Rev Cancer (2018) 18(5):313–22. doi: 10.1038/nrc.2018.6 PMC5912991 29449659

[4] Golden EB Chhabra A Chachoua A Adams S Donach M Fenton-Kerimian M. Local Radiotherapy and Granulocyte-Macrophage Colony-Stimulating Factor to Generate Abscopal Responses in Patients With Metastatic Solid Tumours: A Proof-of-Principle Trial. Lancet Oncol (2015) 16:795–803. doi: 10.1016/S1470-2045(15)00054-6 26095785

[5] Demaria S Coleman CN Formenti SC. Radiotherapy: Changing the Game in Immunotherapy. Trends Cancer (2016) 2:286–94. doi: 10.1016/j.trecan.2016.05.002 PMC5070800 27774519

[6] He S Zhao H Fei M Wu Y Wang L Zhu X. Expression of the Co-Signaling Molecules CD40-CD40L and Their Growth Inhibitory Effect on Pancreatic Cancer *In Vitro* . Oncol Rep (2012) 28(1):262–8. doi: 10.3892/or.2012.1790 22552529

[7] Yasmin-Karim S Bruck PT Moreau M Kunjachan S Chen GZ Kumar R. Radiation and Local Anti-CD40 Generate an Effective *In Situ* Vaccine in Preclinical Models of Pancreatic Cancer. Front Immunol (2018) 9:2030. doi: 10.3389/fimmu.2018.02030 30245691 PMC6137176

[8] Bonaventura P Shekarian T Alcazer V Valladeau-Guilemond J Valsesia-Wittmann S Amigorena S. Cold Tumors: A Therapeutic Challenge for Immunotherapy. Front Immunol (2019) 10:168. doi: 10.3389/fimmu.2019.00168 30800125 PMC6376112

[9] Ochoa de Olza M Navarro Rodrigo B Zimmermann S Coukos G. Turning Up the Heat on non-Immunoreactive Tumours: Opportunities for Clinical Development. Lancet Oncol (2020) 21(9):e419–30. doi: 10.1016/S1470-2045(20)30234-5 32888471

[10] Nair SS Weil R Dovey Z Davis A Tewari AK. The Tumor Microenvironment and Immunotherapy in Prostate and Bladder Cancer. Urol Clinics North Am (2020) 47(4S):e17–54. doi: 10.1016/j.ucl.2020.10.005 33446323

[11] Palmer DH Hussain SA Ganesan R Cooke PW Wallace DM Young LS. CD40 Expression in Prostate Cancer: A Potential Diagnostic and Therapeutic Molecule. Oncol Rep (2004) 12(14):679–82. doi: 10.3892/or.12.4.679 15375484

[12] Ryan DP Hong TS Bardeesy N. Pancreatic Adenocarcinoma. N Engl J Med (2014) 35:353–4. doi: 10.1056/NEJMc1412266

[13] Tang C Wang X Soh H Seyedin S Cortez MA Krishnan S. Combining Radiation and Immunotherapy: A New Systemic Therapy for Solid Tumors? Cancer Immunol Res (2014) 2:831–8. doi: 10.1158/2326-6066.CIR-14-0069 PMC5367158 25187273

[14] Ngwa W Boateng F Kumar R Irvine DJ Formenti S Ngoma T. Smart Radiation Therapy Biomaterials. Int J Radiat Oncol Biol Phys (2017) 97:624–37. doi: 10.1016/j.ijrobp.2016.10.034 PMC5302132 28126309

[15] Mueller R Moreau M Yasmin-Karim S Protti A Tillement O Berbeco R. Imaging and Characterization of Sustained Gadolinium Nanoparticle Release From Next Generation Radiotherapy Biomaterial. Nanomaterials (Basel Switzerland) (2020) 10(11):2249. doi: 10.3390/nano10112249 PMC7697013 33202903

[16] Boateng F Wilfred N. Novel Bioerodable Eluting-Spacers for Radiotherapy Applications With *Insitu* Dose Painting. Br J Radiol (2019) 92:1098. doi: 10.1259/bjr.20180745 20180745. PMC6592086 31084497

[17] Boateng F Wilfred N. Delivery of Nanoparticle-Based Radiosensitizers for Radiotherapy Applications. Int J Mol Sci (2019) 21(1):273. doi: 10.3390/ijms21010273 PMC6981554 31906108

[18] Park J Babensee JE. Differential Functional Effects of Biomaterials on Dendritic Cell Maturation. Acta Biomater (2012) 8(10):3606–17. doi: 10.1016/j.actbio.2012.06.006 PMC3970713 22705044

[19] Chao Y Xu L Liang C Feng L Xu J Dong Z. Combined Local Immunostimulatory Radioisotope Therapy and Systemic Immune Checkpoint Blockade Imparts Potent Antitumour Responses. Nat Biomed Eng (2018) 2(8):611–21. doi: 10.1038/s41551-018-0262-6 31015634

[20] Culig Z Santer FR. Androgen Receptor Signaling in Prostate Cancer. Cancer Metastasis Rev (2014) 33(2-3):413–27. doi: 10.1007/s10555-013-9474-0 24384911

[21] Welsh JS Berta C Borzillary S Sam C Shickell D Nobile L. Fiducial Markers Implanted During Prostate Brachytherapy for Guiding Conformal External Beam Radiation Therapy. Technol Cancer Res Treat (2004) 3(4):359–64. doi: 10.1177/153303460400300405 15270586

[22] Moghaddami M Cohen P Stapleton AM Brown MP. CD40 is Not Detected on Human Prostate Cancer Cells by Immunohistologic Techniques. Urology (2001) 57(3):573–8. doi: 10.1016/s0090-4295(00)01005-0 11248650

[23] Bilusic M Madan RA Gulley JL. Immunotherapy of Prostate Cancer: Facts and Hopes. Clin Cancer Res (2017) 23(22):6764–70. doi: 10.1158/1078-0432.CCR-17-0019 PMC5690854 28663235

[24] Amling CL Bergstralh EJ Blute ML Slezak JM Zincke H. Defining Prostate Specific Antigen Progression After Radical Prostatectomy: What Is the Most Appropriate Cut Point? J Urol (2001) 165:1146–51. doi: 10.1016/S0022-5347(05)66452-X 11257657

[25] Nussbaum N George DJ Abernethy AP Dolan CM Oestreicher N Flanders S. Patient Experience in the Treatment of Metastatic Castration-Resistant Prostate Cancer: State of the Science. Prostate Cancer Prostatic Dis (2016) 19:111–21. doi: 10.1038/pcan.2015.42 PMC4868871 26832363

[26] Tucci M Scagliotti GV Vignani F. Metastatic Castration-Resistant Prostate Cancer: Time for Innovation. Future Oncol (London England) (2015) 11:91–106. doi: 10.2217/fon.14.145 25572785

[27] El-Rayes BF Akce M. Immunotherapy in Pancreatic Cancer. Dig Dis Interv (2020) 04(04):351–7. doi: 10.1055/s-0040-1718904

[28] Ryan DP Hong TS Bardeesy N. Pancreatic Adenocarcinoma. N Engl J Med (2014) 371:1039–49. doi: 10.1056/NEJMra1404198 25207767

[29] Yasmin-Karim S Moreau M Mueller R Sinha N Dabney R Herman A. Enhancing the Therapeutic Efficacy of Cancer Treatment With Cannabinoids. Front Oncol (2018) 2018:114(8). doi: 10.3389/fonc.2018.00114 PMC5928848 29740535

[30] Irabor OC Swanson W Shaukat F Wirtz J Mallum AA Ngoma T. Can the Adoption of Hypofractionation Guidelines Expand Global Radiotherapy Access? An Analysis for Breast and Prostate Radiotherapy. JCO Global Oncol (2020) 6:667–78. doi: 10.1200/jgo.19.00261 PMC7193821 32343628

[31] Bhardwaj A Srivastava SK Khan MA Prajapati VK Singh S Carter JE. Racial Disparities in Prostate Cancer: A Molecular Perspective. Front Biosci (Landmark Ed) (2017) 22:772–82. doi: 10.2741/4515 PMC5242333 27814645

[32] Hao Y Yasmin-Karim S Moreau M Sinha N Sajo E Ngwa W. Enhancing Radiotherapy for Lung Cancer Using Immunoadjuvants Delivered *In Situ* From New Design Radiotherapy Biomaterials: A Preclinical Study. Phys Med Biol (2016) 61(24):N697–707. doi: 10.1088/1361-6560/61/24/N697 PMC5209794 27910826

[33] Kawabe T Matsushima M Hashimoto N Imaizumi K Hasegawa Y. CD40/CD40 Ligand Interactions in Immune Responses and Pulmonary Immunity. Nagoya J Med Sci (2011) 73(3-4):69–78. 21928689 PMC4831216

[34] Bajwa R Cheema A Khan T Amirpour A Paul A Chaughtai S. Adverse Effects of Immune Checkpoint Inhibitors (Programmed Death-1 Inhibitors and Cytotoxic T-Lymphocyte-Associated Protein-4 Inhibitors): Results of a Retrospective Study. J Clin Med Res (2019) 11(4):225–36. doi: 10.14740/jocmr3750 PMC6436564 30937112

[35] Walle T Martinez Monge R Cerwenka A Ajona D Melero I Lecanda F. Radiation Effects on Antitumor Immune Responses: Current Perspectives and Challenges. Ther Adv Med Oncol (2018) 10:1758834017742575. doi: 10.1177/1758834017742575 29383033 PMC5784573

[36] LaVigne AW Triedman SA Randall TC Trimble EL Viswanathan AN. Cervical Cancer in Low and Middle Income Countries: Addressing Barriers to Radiotherapy Delivery. Gynecol Oncol Rep (2017) 22:16–20. doi: 10.1016/j.gore.2017.08.004 28948205 PMC5602511

